# Differentiation and Function of T Cell Subsets in Infectious Diseases

**DOI:** 10.1155/2018/3439025

**Published:** 2018-09-23

**Authors:** Yuejin Liang, Xiaojun Chen, Jinling Chen, Fengliang Liu

**Affiliations:** ^1^Department of Microbiology & Immunology, University of Texas Medical Branch, Galveston, Texas 77550, USA; ^2^Department of Pathogen Biology & Immunology, Jiangsu Key Laboratory of Pathogen Biology, Nanjing Medical University, Nanjing, Jiangsu 210019, China; ^3^Department of Pathogen Biology, School of Medicine, Nantong University, Nantong, Jiangsu 226001, China

Infectious diseases remain a public health problem in the world, regardless of the continued effort at control. The aim of the host immune response during infection is to clear invading pathogens with limited tissue damage. Both innate and adaptive T cells play a key role in direct pathogen clearance through proinflammatory cytokine and cytotoxic T lymphocyte (CTL) activity. In addition, T helper (Th) cells and regulatory T (Treg) cells are required for plasma cell-secreted antibodies and immunomodulatory cytokines (*e.g.*, IL-10), respectively. In recent years, the role of novel Th cell subsets, including Th17, Th22, and T follicular cells, in regulating anti-infectious immunity, has gained much importance, since they play a crucial role in the development and outcome of diseases. In addition to adaptive T cells, there are unconventional T cells, such as *γδ* T cells and NKT cells, which are defined as different subpopulations (*e.g*., IFN-*γ*^+^*γδ* T cells vs. *γδ*17 T cells and IFN-*γ*^+^ NKT cells vs. NKT17 cells) based on their cytokine production signatures. These innate-like T cells rapidly respond to pathogens and display effector functions without undergoing extensive clonal expansion. An understanding of T cell differentiation and function in infectious diseases, as well as their underlying mechanisms, may contribute to the development of potential therapies for emerging infectious diseases in the future.

In this special issue, a total of 15 manuscripts were received and eight manuscripts have been accepted for publication after several rounds of review. Three of these publications are review articles that provide comprehensive information regarding the role of innate and adaptive T cells in infectious diseases. R. Zheng et al. generated a meta-analysis by searching published articles identified as relating to the clinical features of human brucellosis in China. They described a significant decrease of CD4^+^, but increase of CD8^+^ T cells, as well as a reduction of CD4^+^/CD8^+^ ratio in the blood of human brucellosis patients compared to healthy subjects. D. A. Cronkite and T. M. Strutt focus on the regulation of inflammation by lymphocytes. The recent advancements in our understanding of inflammatory triggers, imprinting of the innate immune responses, and the role of T cell memory in regulating inflammation are discussed in this review paper. In particular, the field of innate immune cell memory must be further investigated in order to effectively implement this new insight into vaccine design and clinical therapy for infectious diseases. Y. Zhao et al. explore the role of *γδ* T cells in different infections and their potential application in the clinic. They focus on various subsets of *γδ* T cells, which play a critical role in regulating host immunity against pathogens, including bacteria, viruses, and parasites. Since *γδ* T cells are involved in the elimination of pathogens, this cell type might have promising implications for the treatment of infectious diseases in preclinical studies.

Among the five research articles in this special issue, three manuscripts from Chinese research groups are associated with *Schistosoma* or *Schistosoma*/HIV coinfection. Y. Zhu et al. demonstrated that the administration of pioglitazone, which is an agonist of peroxisome proliferator-activated receptor- (PPAR-) *γ*, reduces splenic and hepatic immunopathogenesis in *Schistosoma japonicum*-infected mice through inducing Treg cells. Moreover, PPAR-*γ*agonist can promote Foxp3 expression through both macrophage-dependent and independent manners. Y. Yang et al. demonstrate that the CD4^+^/CD8^+^ T cell ratio is lower in *Schistosoma*/HIV coinfection patients compared with levels in patients with HIV- or *Schistosoma*-infection only. This result suggests significant immune suppression in coinfection patients. X. Chen et al. determine that Good's syndrome (GS) patients have an inverted ratio of CD4^+^/CD8^+^ T cells, more Treg cells, and a lower percentage of V*γ*2 subpopulation in *γδ* T cells in the blood. In particular, CD4^+^ T cells from GS patients show an insufficient ability to proliferate and express higher levels of PD-1, explaining the impaired CD4^+^ T cell responses in GS patients.

There are two manuscripts that are not directly linked to the study of T cells but highlight the important immune components that can regulate T cell responses. R. C. Araújo et al. detected an increased level of human leukocyte antigen E (HLA-E) in hepatocytes and Kupffer cells of HCV patients compared with that in health people. Since HLA-E can bind and present peptides to antigen-specific CD8^+^ T cells, it may play a role in regulating T cell activation in HCV patients. J. Qu et al. underscore the immunomodulatory role of Toll-like receptor (TLR3) in NK cells. They demonstrate that TLR3^+^ NK cells display activated phenotypes as evidenced by upregulated surface activation markers and increased cytokine production. Although TLR3 expression is comparable on T cells after *Schistosoma* infection, TLR3-dependent NK cell activation may regulate T cell responses through cell-cell interaction and the cytokine microenvironment in lymphoid organs.

In summary, this special issue covers several important aspects of T cell functions related to infectious diseases. We hope that these articles can provide guidance for further research on T cell functions and effective therapeutics.

